# Sex differences in the expression of cell adhesion molecules on microvesicles derived from cultured human brain microvascular endothelial cells treated with inflammatory and thrombotic stimuli

**DOI:** 10.1186/s13293-019-0241-y

**Published:** 2019-05-22

**Authors:** Larry W. Hunter, Muthuvel Jayachandran, Virginia M. Miller

**Affiliations:** 1 Department of Surgery, Mayo Clinic, Medical Science Bldg. 4-20, 200 First St. SW, Rochester, MN 55905 USA; 2 Department of Physiology and Biomedical Engineering, Mayo Clinic, Rochester, MN 55905 USA; 3 Divisions of Hematology Research and Nephrology and Hypertension Research, Mayo Clinic, Rochester, MN 55905 USA; 4 Women’s Health Research Center, Mayo Clinic, Rochester, MN 55905 USA

**Keywords:** Cerebrovascular, Blood-brain barrier, Cell adhesion molecules, Extracellular vesicles, Sex differences

## Abstract

**Background:**

There are sex differences in risk for stroke and small vessel ischemic disease in the brain. Microvesicles (MV) derived from activated cells vary by cell of origin and the stimulus initiating their release. MV released from cells activated by inflammatory and thrombotic factors have the potential to disrupt endothelial cells of the brain microvasculature. Therefore, experiments were designed to identify sex differences in the phenotype of MV released from cultured human brain microvascular endothelial cells (HBMEC) in response to inflammatory and thrombotic stimuli.

**Methods:**

Cultured HBMEC derived from 20- to 30-year-old male and female donors were treated for 20 h with medium supplemented with tumor necrosis factor alpha (TNFα; 20 ng/ml), thrombin (THR; 2 U/ml), or vehicle (i.e., control). MV were isolated from the conditioned media by high-speed centrifugation and quantified by digital flow cytometry by labeling with fluorophore-conjugated primary antibodies against PECAM-1, integrin αvβ3, ICAM-1, E-selectin, or MCAM. In addition, temporal uptake of labeled MV into control HBMEC was examined by confocal microscopy.

**Results:**

Under control conditions, male HBMEC released fewer MV expressing each antigen, except for PECAM-1, than female cells (*P* < 0.05). Neither TNFα nor THR reduced cell viability. However, TNFα induced apoptosis in female and male cells, whereas THR increased apoptosis marginally only in male cells. TNFα increased expression of all antigens tested on MV in male cells, but only increased expression of integrin αvβ3, ICAM-1, and E-selectin on MV from female cells. THR increased expression of PECAM-1, ICAM-1, and MCAM-1 on MV from male but not female cells. MV were internalized and localized to lysosomes within 90 min after their application to HBMEC.

**Conclusions:**

There are sex differences in expression of cell adhesion molecules on MV released from HBMEC under control conditions and upon activation by TNFα or THR. MV taken up by unstimulated HBMEC may impact the integrity of the brain microvasculature and account, in part, for sex differences in vascular pathologies in the brain.

## Background

Dysfunction of vascular endothelial cells (ECs) is considered an early event in the development of chronic vascular diseases [1], in acute vascular events following traumatic brain injury [2] and ischemic stroke [3], and in development of Alzheimer’s disease [4, 5]. There are sex differences in outcomes from various pathologies involving the cerebral vasculature [6–10]. In the brain, these vascular pathological conditions are closely associated with several endothelial processes including release of endothelium-derived microvesicles (MV) [3, 11].

MV, also referred to as microparticles, are submicron- sized membrane-encapsulated structures that are shed from the plasma membrane of activated cells and which mediate cell to cell communication by transporting cell adhesion molecules on their surface, as well as encapsulated cell proteins, DNA, RNA, enzymes, lipids, and metabolites to the target cells [12, 13]. The phenotype of MV released from activated cells contains overlapping as well as distinct protein compositions, depending upon the cell of origin as well as the stimulus which initiated their generation [14, 15]. For example, platelet endothelial cell adhesion molecule-1 (PECAM-1), integrin αvβ3, intercellular adhesion molecule-1 (ICAM-1), E-selectin, and melanoma cell adhesion molecule/cell surface glycoprotein MUC18 (MCAM) are upregulated in human cerebrovascular endothelial cells under inflammatory conditions and mediate a multi-step process resulting in transmigration of immune cells into the brain [16–20]. E-selectin, which is expressed specifically on activated endothelial cells, is rapidly induced and binds with low affinity to ligands on various cell types for their recruitment to sites of inflammation. PECAM-1, ICAM-1, and MCAM mediate leukocyte activation and their adhesion to vascular endothelium, integrin activation, and angiogenesis. And in the brain, integrin avβ3 anchors endothelial cells to matrix components, and to other cell types, and mediates cytoskeletal organization of cells of the blood-brain barrier [17].

In vascular diseases, activation of endothelial cells by thrombotic and inflammatory cytokines, hormones, shear, or oxidative stress induces release of endothelium- derived vasoactive factors and MV into the circulation, the amounts of which may associate with the severity of the disease [19, 21–23]. In vitro, activated endothelial cells also release MV with distinctive array of antigens, depending upon the particular vascular bed origin [24, 25]. Tumor necrosis factor alpha (TNFα) and thrombin (THR) initiate MV release through cell membrane receptors TNF receptor-1 (TNFR1) and protease-activated receptor-1 (PAR-1), respectively, to produce proinflammatory/procoagulant MV [26]. There are sex differences in circulating MV from healthy humans [27], in cerebrovascular function, and in stroke outcomes [28, 29]. However, little experimental evidence exists regarding sex differences in release or characterization of MV associated with the cerebral microvasculature [8, 30]. Thus, this study was designed to determine if there are sex differences (1) in viability of brain microvascular endothelial cells stimulated with inflammatory and thrombotic stimuli (TNFα and THR, respectively) and (2) in the expression of surface antigens on the MV released from the stimulated cells. Proof-of-concept experiments were performed to determine the time course of MV uptake by endothelial cells and their disposition within the cells [31].

## Materials and methods

### Materials, antibodies, and reagents

Human brain microvascular endothelial cells (HBMEC) used in these experiments were from a Caucasian male (21 years of age; cause of death—car accident; Neuromics, Edina, MN, USA: HEC02; lot HECO208032015) and a Caucasian female (26 years of age; cause of death—flail chest injury from an automobile accident; iXCells Biotechnologies, San Diego, CA, USA: 10HU-051; lot 200,139). Endothelial cell medium (ECM), fetal bovine serum (FBS), endothelial cell growth supplement (ECGS), and recombinant human TNFα were from ScienCell, Carlsbad, CA, USA. Antibiotic- antimycotic supplement, gelatin attachment factor, ProLong™ Gold cell mounting medium with 4′,6-diamidino-2-phenylindol (DAPI), and phosphate- buffered saline (PBS) were from Thermo Fisher Scientific, Waltham, MA, USA. Fluorescein isothiocyanate- conjugated annexin V (annexin V-FITC) and phycoerythrin (PE)- conjugated antibodies against platelet endothelial cell adhesion molecule-1 (PECAM-1), integrin αvβ3, intercellular adhesion molecule-1 (ICAM-1), E-selectin, and melanoma cell adhesion molecule/cell surface glycoprotein MUC18 (MCAM) for digital flow cytometric analysis (FACS) were obtained from BD Biosciences, San Jose, CA, USA, and CellTiter96® cytotoxicity assay was from Promega, Madison, WI, USA. PKH67 fluorescent cell membrane dye and early endosome antigen-1 (EEA-1) mouse monoclonal antibody were from Sigma- Aldrich, St. Louis, MO, USA, and Santa Cruz Biotechnology, Inc., Santa Cruz, CA, USA, respectively. LysoTracker™ Red (LTR) and AlexaFluor 647 goat anti-mouse secondary antibody were from Thermo Fisher Scientific.

### HBMEC culture

HBMEC at passage 3 or 4 were suspended in ECM containing ECGS, antibiotic-antimycotic, and 10% fetal bovine serum (FBS) and plated at a density of 2.5 × 10^3^ cells/cm^2^ onto gelatin-coated surfaces, then incubated at 37 °C, with 5% CO_2_. The medium was replaced every 2 days, and the cells were used for experimentation when 90–95% confluent.

### Cell viability and apoptosis assays

Cell viability was determined colorimetrically by CellTiter96® cell proliferation assay, using the dehydrogenase substrate 3-(4,5-dimethylthiazol-2-yl)-5-(3-carboxymethoxyphenyl)-2-(4-sulfophenyl)-2H-tetrazolium; MTS. Cells were plated into a 96-well plate, cultured to about 90% confluency, then treated with fresh medium containing either TNFα (20 ng/ml), THR (2 U/ml), or vehicle (control) for 20 h. Staurosporine (0.5 μM) was used concurrently as a positive control. Twenty microliters of MTS was then added to each well, and after 3 h at 37 °C, 490 nm absorbance of the formazan product was measured using a microplate reader (Clariostar; BMG Labtech, Cary, NC, USA). The quantity of product measured by this method is directly proportional to the number of living cells.

Apoptosis was determined by measurement of cleavage of methylcoumarin (Ac-DEVD-AMC), a fluorogenic caspase-3-specific substrate, in cell lysates after experimentation. Briefly, cells were cultured in 24-well plates, treated as described above, then rinsed with PBS, and lysed in 150 μl of CHAPS lysis buffer on ice. Fifty microliters of lysate was added to 150 μl of HEPES reaction buffer containing 10 μM (final concentration) of the substrate. After 45-min incubation at 37 °C, the liberated fluorescent product was measured at 380/465 nm using a microplate assay. Staurosporine effects in vitro are concentration-dependent; therefore, to use this agent as a positive control to assess apoptosis, the concentration was reduced to 0.1 μM [32].

### Generation and characterization of MV

HBMEC were seeded into 25-cm^2^ flasks and cultured for 4 days. Medium was then removed, and each flask was rinsed 3 times in serum-free medium before adding culture medium containing 5% FBS with either vehicle (control), TNFα (20 ng/ml), or THR (2 U/ml). Before use, the FBS was cleared of possible MV contamination by centrifugation at 20,000×*g* for 30 min, followed by 0.1 μm membrane filtering. A flask containing medium without cells was also examined as a negative control.

The purpose of these experiments was to characterize antigen expression on MV derived from endothelial cell plasma membranes. Therefore, after 20-h incubation, the conditioned medium was removed and centrifuged at 2000×*g* for 10 min to remove cellular debris or fragments, detached, or dead cells. The supernatant was then centrifuged at 20,000×*g* for 30 min as described previously for plasma MV isolation [33]. The pelleted MV were suspended in serum-free medium by vortexing for 1–2 min and centrifuged at 20,000×*g* for 30 min. The final pellets were suspended in original volume of serum-free medium and vortexed for 1–2 min. The method of isolation was adopted from our previous publications for pelleting of larger size vesicles such as microvesicles from platelet-free plasma and cell-free urine [33–35].

MV in 50 μl aliquots were labeled with annexin V-FITC, paired with a PE-conjugated antibody against either PE CAM-1, integrin avβ3, ICAM-1, E-selectin, or MCAM, then quantified by FACS (FACSCanto™) with a size > 150 nm as described previously [33, 36]. The total numbers of each MV antigenic phenotype per flask of conditioned medium were determined. The fold increase in number above control (unstimulated) conditions was determined for each adhesion molecule and stimulus.

### MV uptake into HBMEC

MV derived from untreated female cells were isolated as described above and quantified by FACS for total PECAM-1/annexin V-positive vesicles, then labeled with PKH67, a green fluorescent cell membrane marker, according to the manufacturer’s protocol. The MV (1000 MV/μl final concentration) were then applied to confluent, previously unstimulated female cells cultured on glass cover-slips for 30 min, 90 min, or 20 h. Non- MV-treated cells served as a control. At each time point, duplicate cover-slips were rinsed in fresh medium then the adhered cells were labeled with markers for intracellular structures. First, LTR (50 nM final concentration), to label lysosomes, was applied for 30 min. Then, after rinsing, the cells were fixed for 10 min in 4% paraformaldehyde and permeabilized in 0.1% Triton X-100 for 10 min. After rinsing again in PBS, the cells were incubated overnight at 4 °C with EEA-1 mouse monoclonal antibody to label early endosomes. After rinsing, Alexa Fluor 647 secondary antibody was applied for 1 h. Finally, the samples were rinsed, then mounted on glass slides, using mounting medium containing DAPI (4′,6-diamidino-2-phenylindole) to label nuclei. Specimens were examined using a Zeiss LSM780 confocal laser- scanning microscope fitted with a Zeiss 63X water- immersion lens. For each random field examined, 12 optical slices were collected and used to generate a maximum intensity projection for analysis. All images were collected using sequential scanning of individual fluorescence channels, to reduce the likelihood of false co-labeling.

### Statistical analysis

Data are presented as mean ± standard error of the mean (SEM) of 4 or 5 experiments for each study. Differences between treatments of the same donor cells were examined using the two-tailed paired *t* test, and differences between male and female cells for each parameter were examined using the two-sample *t* test with equal variance. Differences were considered significant at *P* < 0.05.

## Results

### Treatment effects on cell viability and apoptosis

**Fig. 1:**
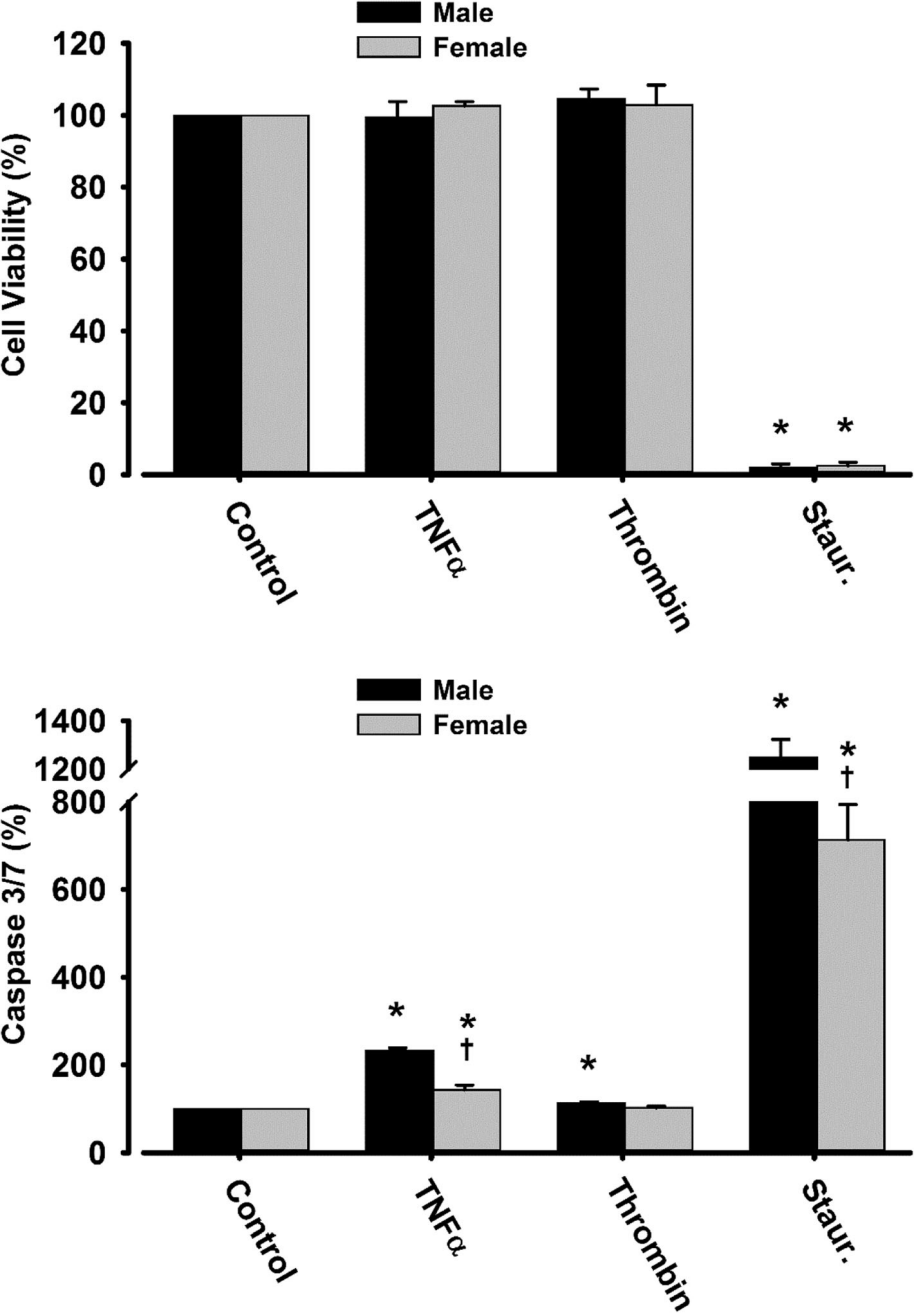
Cell viability (upper panel) determined by analysis of dehydrogenases of metabolically active cells and apoptosis (lower panel) defined by cleavage of the caspase-3-specific substrate Ac-DEVD-AMC in HBMEC under unstimulated conditions (control) or following treatment with TNFα (20 ng/ml), THR (2 U/ml), or staurosporine (Staur), as a positive control (0.5 μM for upper panel and 0.1 μM for lower panel) for 20 h. Data are presented as mean ± SEM. *P < 0.05: vs same sex untreated, controlled cells, by paired *t* test; †, vs same parameter in male cells, by two-tailed *t* test. *N* = 4 for each condition

MV were not detected in culture medium without cells. Under the conditions used to generate MV, neither TNFα nor THR treatments altered cell viability compared to vehicle-treated cells, although viability was significantly reduced by staurosporine, a positive control (Fig. 1, upper panel). Apoptosis, measured by cleavage of the caspase- 3-specific substrate Ac-DEVD-AMC, was significantly increased by treatment with TNFα in male and female cells, but the increase was greater in male compared to female cells (Fig. 1, lower panel). Treatment with THR modestly increased caspase-3 activity in male cells. Staurosporine significantly increased caspase-3 activity in both male and female cells (Fig. 1, lower panel).

### Characterization of MV

**Fig. 2:**
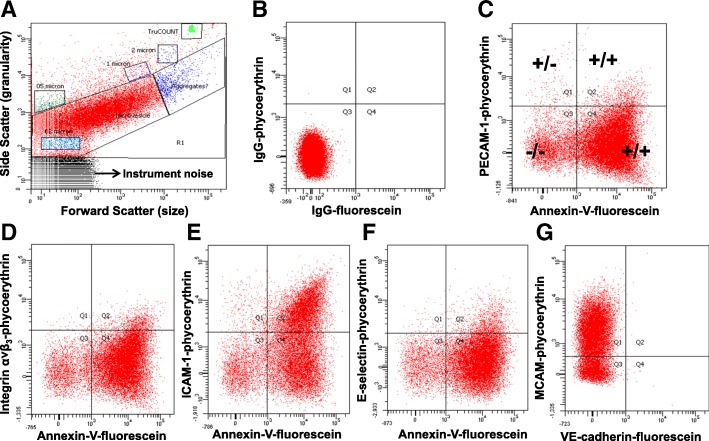
An example of a light scatter plot (**a**) and fluorescence dot plots (**b**–**g**) derived from the microvesicle (0.2–1 μm size) gate of the digital flow cytometer analysis of cell-free culture media from TNFα (20 ng/ml) stimulated HBMEC for 20 h. The size gates were determined based on calibration size (0.2–2 μm) beads and with truCOUNT™ (4.2 μm size) beads (**a**). The positive and negative counts of microvesicles were separated by fluorescence dot plots derived from microvesicle gate of scatter plot of phycoerythrin- and fluorescein (FITC)-conjugated isotype control antibodies (**b**) and selected candidate cell membrane marker antibodies (**c**, PECAM-1; **d**, Integrin α˅β_3_; **e**, ICAM-1; **f**, E-selectin) plus recombinant annexin-V for surface phosphatidylserine and MCAM plus VE-cadherin (**f**) antibodies stained microvesicles. The example separation of single (+/− (Q1) or −/+ (Q4)), double (+/+ (Q2)) marker positive, and both marker negative (−/− (Q3)) microvesicles is shown in **c**

**Fig. 3:**
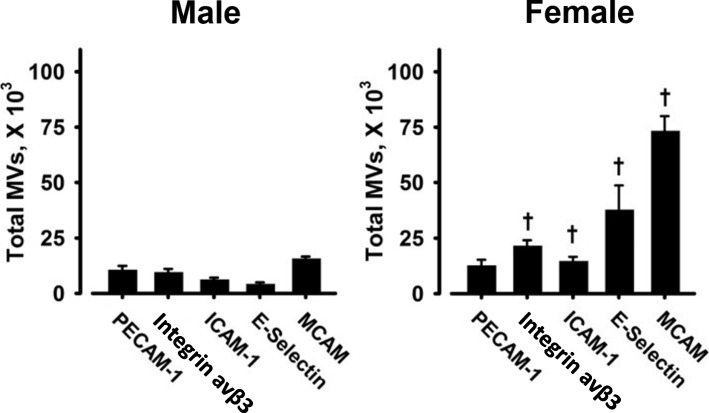
Total numbers of each MV phenotype in conditioned media from cultured male (left panel) and female (right panel) HBMEC treated for 20 h with medium without stimulant and used for baseline control values. Data are shown as mean ± SEM (*n* = 4–5 experiments per each condition) of total MV in conditioned media. †, *P* < 0.05, different from media conditioned by male cells

**Fig. 4:**
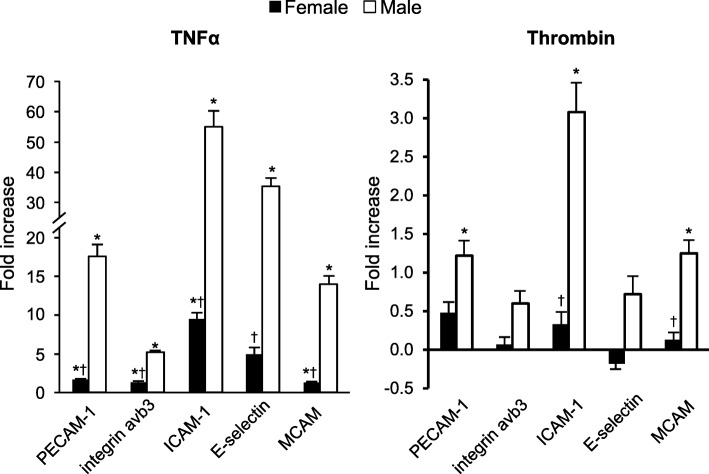
Fold increase in release of MV from male (open bars) and female (black bars) HBMEC expressing cell adhesion molecules following stimulation with either TNFα (20 ng/ml) or THR (2 U/ml) for 20 h. Data are shown as mean ± SEM fold increase above the number of MV expressed under unstimulated (control) conditions from 4 to 5 separate sets of experiments per condition. *P < 0.05 vs same sex untreated cells, by paired *t* test; †, vs same parameter in male cells, by two-tailed *t* test

Representative FACS analysis of fluorescence dot plots of PECAM-1-expressing MV released from male HBMEC under control, unstimulated conditions and following stimulation with TNFα (20 ng/ml) for 20 h is shown in Fig. 2. The total number of each MV phenotype under control conditions ranged from 4.0 ± 1.0 to 15.5 ± 1.1 × 10^3^ in media conditioned by male cells and from 12.4 ± 2.9 to 73.1 ± 6.9 × 10^3^ in media conditioned by female cells. Under control conditions, male HBMEC released fewer antigen- positive MV than did female cells, with the exception of PECAM-1-positive MV which were similar in both groups (Fig. 3). In media conditioned by male cells, there was a significant fold increase above control conditions in MV expressing each of the antigens following treatment with TNFα, and in media conditioned by female cells, increases were observed for all antigen phenotypes, except E-selectin (Fig. 4). Further, fold increase in MV positive for all antigens was greater in media conditioned by male compared to female cells following treatment with TNFα (Fig. 4). THR treatment increased MV expressing PECAM-1, ICAM-1, and MCAM in media conditioned by male cells, but was without effect on female cells (Fig. 4). In addition, the fold increase in MV expressing ICAM-1 and MCAM was greater in media conditioned from male compared to female THR-treated cells (Fig. 4).

### Uptake of MV into HBMEC

**Fig. 5:**
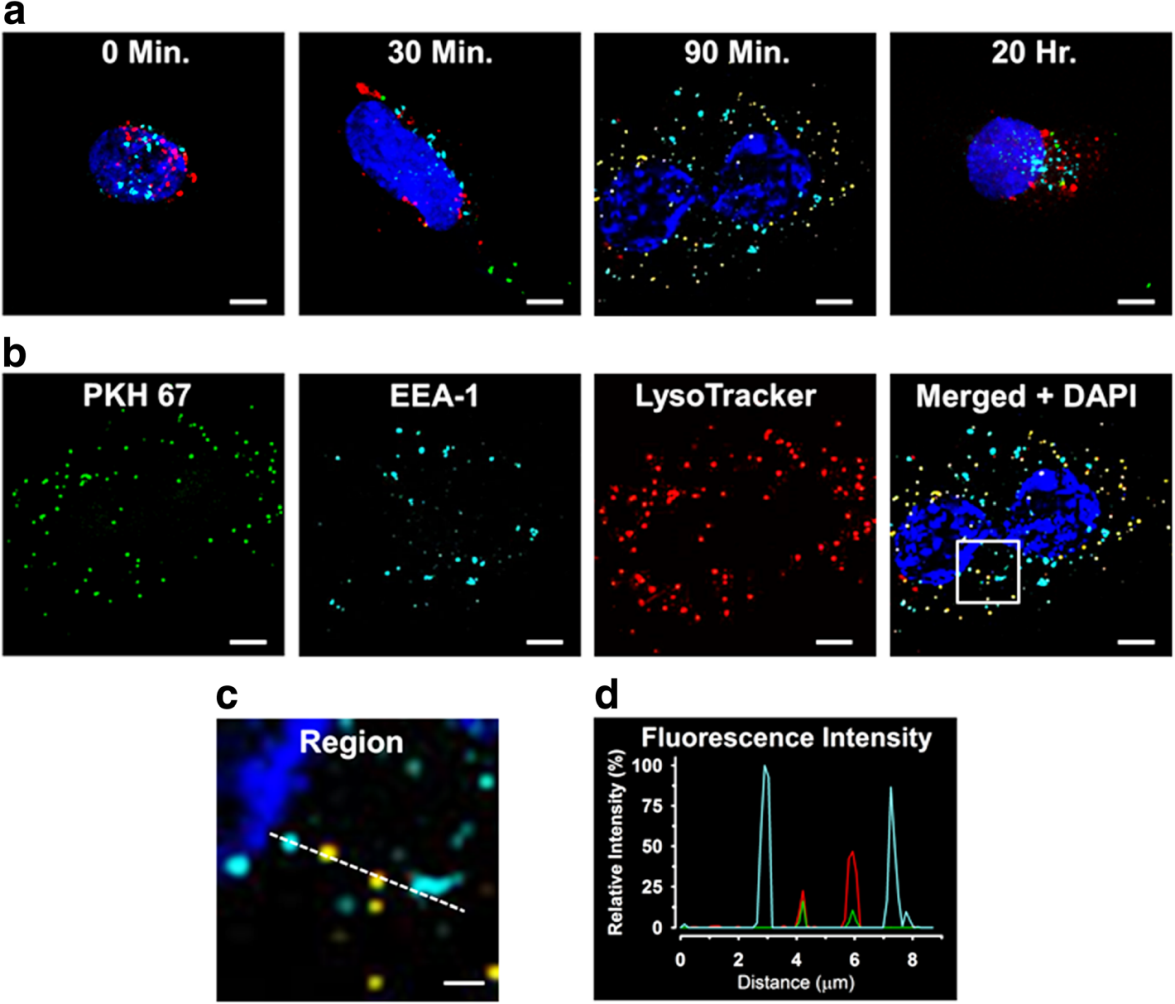
Confocal micrographs of internalization of MV derived from unstimulated HBMEC by sex-matched unstimulated HBMEC. MV generated from female donor cells were first labeled with PKH67 (green), then rinsed and applied to naïve female recipient cells for various time intervals; non-MV-treated cells served as controls. At each time point, 30 min, 90 min, or 20 h, paired sets of control and MV-exposed cells were rinsed to remove non-internalized MV and labeled with markers for (1) early endosome antigen-1 (EEA-1) conjugated with IgG/AlexaFluor 647 IgG (cyan), (2) lysosomes [LysoTracker™ (red)], and (3) nuclei [DAPI (blue)] as described in the “Materials and methods” section. **a** Merged maximal intensity projections of cells not treated (0 min) or treated with PKH67-labeled MV for 30 min, 90 min, or 20 h. Sparse PKH67-positive punctate structures were present in the cytoplasm 30 min after MV application and were increased in number after 90 min, most of which were co-labeled for lysosomes (yellow). MV labeling was reduced at 20 h. Co-localization of MV/early endosome labels was absent at all time points examined. **b** The image of the 90-min MV-treated cells (above) was used to substantiate accurate assessment of localization of each fluorescent marker. Images obtained by sequential scanning of individual channels for PKH67, EEA-1, and LTR are shown, together with the resulting merged image which also includes the DAPI channel. The region bounded by white box in the merged image from 90 min was enlarged (**c**), and a representative fluorescence intensity profile along the dashed line encompassing labeled cytoplasmic structures showed overlapping signals for PKH67 with LTR, but not with early endosomes (**d**). White bars, 5 μm; except in **c**, 1 μm

Following the 30-min incubation period with PKH67- labeled MV derived from untreated female donor HBMEC, sparse cytoplasmic punctate structures which labeled positively for PKH67 (green) were observed within the treated cells. Co-labeling of PKH67 with the early endosome (EEA-1, cyan) or lysosome (LTR, red) markers was absent (Fig. 5a). PKH67 labeling within the treated cells increased after 90 min and was almost entirely co-localized with lysosomes, indicated by yellow staining. Except for DAPI (blue), all labeling was markedly reduced after exposure to the labeled MV for 20 h. Co-localization of MV with the early endosome label was absent at all time points.

In other experiments, cells were incubated with labeled MV for 90 min to substantiate the methodology used to identify cellular localization of the fluorophores. After incubation, the cells were imaged as described above, then the individual maximal intensity projections obtained in each fluorescent channel were compared, which showed similar labeling patterns for PKH67 together with LTR, but not with EEA-1 IgG/AlexaFluor 647 IgG (Fig. 5b). These results were reinforced by analysis of the corresponding merged image of all channels, using line fluorescence intensity profiles of cell structures encompassing all labels (Fig. 5c) which demonstrated that MV labeling was co-localized with lysosomes, but not with early endosomes (Fig. 5d).

## Discussion

Results of this study demonstrate that there were no differences in cell viability but there were distinct sex differences in adhesion molecules expressed on MV released from cultured HBMEC in response to inflammatory and thrombotic stimuli. The experimental conditions used to generate MV in this study resulted in specific patterns of cellular activation to each stimulus, an observation that is consistent with previous findings [24, 25, 37]. Although neither TNFα nor THR treatment changed cell viability, these inflammatory and thrombotic stimuli caused modest increases in cell caspase-3 activity indicating that a small proportion of the cells were in the early stages of apoptosis. The larger increase in apoptosis observed in male compared to female cells in response to TNFα is consistent with other observations of divergent cellular pathways associated with cell survival in stroke [6, 38].

MV were recovered in medium from untreated cells, most likely indicating spontaneous release of MV during cellular homeostatic processes and is consistent with detection of the presence of low number of endothelium-derived MV in the circulation of healthy humans [27]. Except for PECAM-1, under control condition, there were greater numbers of MV in conditioned media derived from female than from male cells, perhaps reflecting differences in glycocalyx structure or cellular metabolism [39, 40].

Sex differences in MV phenotypes were also observed following stimulation with TNFα and THR. Treatment of male donor cells with TNFα increased expression of cell adhesion molecules on the released MV, with the fold increase being the greatest in ICAM-1 and E-selectin phenotypes. A similar pattern for ICAM-1 and E-selectin MV was observed in female-derived MV, although the sensitivity to TNFα was markedly reduced compared to that of male cells. Among the cellular adhesion molecules examined, ICAM-1 and E-selectin are inducible molecules, which are upregulated within several hours in brain microvascular endothelial cells activated by cytokines, lipopolysaccharide, and thrombin in vitro [24, 25, 37, 41, 42]. In the present study, THR treatment increased expression of ICAM-1, PECAM-1, and MCAM on MV from male cells, although in numbers substantially lower than following treatment with TNFα. In contrast, THR treatment was ineffective in altering MV release from female cells. Thrombin increases release of nitric oxide and other endothelium- derived factors from endothelial cells [43–45]. However, relationships among release of endothelium-derived vasoactive factors and formation and release of MV are unclear.

Confocal microscopy showed that MV applied to otherwise untreated cells are internalized within 30 min. Co-localization of MV labeled with the EEA-1 antibody, which identifies early endosomes, was not observed, suggesting that the MV were within endocytotic vesicles at this time point. However, by 90 min, MV-positive compartments within the cytoplasm increased in number, almost all of which were localized to lysosomes. These results demonstrate that the MV were internalized by HBMEC and trafficked to lysosomes which were degraded by 20 h. The internalization of MV formed by autologous cells, that is, the cell of origin, in response to cell stimulation remains to be determined.

Taken together, these findings provide insight into sex differences in MV formation in HBMEC under basal and stimulated conditions. The inducible molecules ICAM-1 and E-selectin, which are principal effectors of leukocyte recruitment and trafficking into the brain, were the most responsive MV antigens and predominated in male cells [18]. All adhesion molecules examined in this study have been associated with pathological conditions which promote deficiencies in the brain microvasculature including the blood-brain barrier [17–20]. The present results are consistent with a regulatory function for MV derived from the human brain microvasculature in inflammatory and neurodegenerative disorders, traumatic brain injury, and stroke and suggest that they may be partly responsible for sex-specific differences observed in these pathologies.

This study has several limitations. The male and female brain microvascular cells used in these experiments were purchased from two different companies. To control against batch to batch variability and suppliers, all experiments for this study were conducted on cells from the same sources of male and female cells. The donors were of similar age and their death an immediate result of an automobile accident, and not a chronic illness, suggesting that the donors had been in good health. It is recognized that culture conditions such as media and flask/slide material can affect the outcome of these types of experiments. All experiments were performed in media that was not stripped of hormones so as to maintain the cultures. Thus, how the generation of MV might be affected by specific sex steroid hormones in female and male cells is open to investigation. However, given these limitations and under standardized experimental conditions, the results point to sex differences in generation of MV in response to inflammatory stimuli. These differences in responsiveness between male and female endothelial cells could contribute to changes in integrity of the brain microvasculature associated with sex differences in outcomes of stroke and perhaps processes associated with cognitive decline.

## Conclusions

The expression of cell adhesion molecules on human brain microvascular endothelium-derived MV under the control, unstimulated conditions and during activation by TNFα or THR differs by the sex of the microvascular endothelial cells. MV have the capacity to modulate the function of other cells potentially impacting immune cell adhesion and migration, or barrier competency; thus, these differences in MV phenotypes may account, in part, for sex differences in vascular pathologies involving the brain, as well as in normal vascular development and aging. The findings also underscore the appropriateness of identifying the sex of cells used in research studies of MV disposition within the vasculature, as well as the sex of cells used to generate MV for in vitro studies. Future studies are needed to determine how sex hormones affect the release of MV from these cells.
